# Spatio-Temporal Mapping of L-Band Microwave Emission on a Heterogeneous Area with ELBARA III Passive Radiometer †

**DOI:** 10.3390/s19163447

**Published:** 2019-08-07

**Authors:** Łukasz Gluba, Mateusz Łukowski, Radosław Szlązak, Joanna Sagan, Kamil Szewczak, Helena Łoś, Anna Rafalska-Przysucha, Bogusław Usowicz

**Affiliations:** Institute of Agrophysics, Polish Academy of Sciences, Doświadczalna 4, 20-290 Lublin, Poland

**Keywords:** L-band radiometry, microwave remote sensing, soil moisture, electromagnetic moisture sensors, ELBARA

## Abstract

Water resources on Earth become one of the main concerns for society. Therefore, remote sensing methods are still under development in order to improve the picture of the global water cycle. In this context, the microwave bands are the most suitable to study land–water resources. The Soil Moisture and Ocean Salinity (SMOS), satellite mission of the European Space Agency (ESA), is dedicated for studies of the water in soil over land and salinity of oceans. The part of calibration/validation activities in order to improve soil moisture retrieval algorithms over land is done with ground-based passive radiometers. The European Space Agency L-band Microwave Radiometer (ELBARA III) located near the Bubnów wetland in Poland is capable of mapping microwave emissivity at the local scale, due to the azimuthal and vertical movement of the horn antenna. In this paper, we present results of the spatio-temporal mapping of the brightness temperatures on the heterogeneous area of the Bubnów test-site consisting of an area with variable organic matter (OM) content and different type of vegetation. The soil moisture (SM) was retrieved with the L-band microwave emission of the biosphere (L-MEB) model with simplified roughness parametrization (SRP) coupling roughness and optical depth parameters. Estimated soil moisture values were compared with in-situ data from the automatic agrometeorological station. The results show that on the areas with a relatively low OM content (4–6%—cultivated field) there was good agreement between measured and estimated SM values. Further increase in OM content, starting from approximately 6% (meadow wetland), caused an increase in bias, root mean square error (RMSE), and unbiased RMSE (ubRMSE) values and a general drop in correlation coefficient (R). Despite a span of obtained R values, we found that time-averaged estimated SM using the L-MEB SRP approach strongly correlated with OM contents.

## 1. Introduction

Water is one of the most critical resources on Earth. It forms the global climate and supports the life evaluation on Earth as well as determines the ability for food production as a component in the photosynthesis process. Although water contained in soil is approximately only 0.001% of accessible water resources, its presence as soil moisture (SM) is of significant importance. Unfortunately, human activities, as well as increasing population, contribute to an excessive reduction of the water resources. Thus, global monitoring of the parameter concerning water resources is necessary. In order to improve the study on the water cycle at the global prospect, the air- and space-borne remote sensing methods have been developed [1,2,3,4]. For SM sensing, the satellite systems operate at the microwave frequencies range below the relaxation frequency of the free water molecule [5]. The frequencies that compromise between low atmospheric opacity, achievable satellite’s antenna size, and relatively low level of radio frequency interference (RFI) attenuation of the electromagnetic waves by plants lie in microwave L-band, i.e., 1–2 GHz range. The Soil Moisture and Ocean Salinity (SMOS) satellite developed by the European Space Agency (ESA) provides global maps of the L-band (1.4 GHz) microwave brightness temperatures (*T*_B_) [4]. Another relevant L-band satellite is the Soil Moisture Active Passive (SMAP) launched by NASA in 2015 [6]. Since SMOS was designed as a passive instrument, the idea of the SMAP assumes to combine advantages of passive and active sensors in order to decrease the pixel size (unfortunately the active sensor failed during the mission). In parallel, for good agreement between calculated and real SM values the SMOS and SMAP Earth observation missions are supported by ground-based validation/calibration measurements. Up to now, much endeavor was put to gain a better understanding of the L-band microwave emission from soils. The research includes the investigation of the influence of parameters such as vegetation, roughness, or organic matter fraction in soil on SM retrievals. For this purpose, campaigns with tower-based passive radiometers for SMAP and SMOS space-borne missions were conducted [7,8,9,10,11,12,13,14,15,16,17,18]. Currently, the SM retrieval algorithms are calibrated for mineral soils despite a small amount of organic matter can significantly change its hydraulic and dielectric properties. Dielectric mixing model for SM retrieval in SMOS considers only clay content, as a fraction that modifies water holding capacity in mineral soil [19] compromising global availability of data. Nevertheless, there are attempts to incorporate a parametrization associated with organic matter content into the retrieval algorithm [20]. Microwave emission from natural organic soil investigated using tower radiometers has not been sufficiently investigated yet. Recently, L-band microwave emission studies were performed for soil with an organic matter content of 16%: Uniquely rich in organic matter soil from Denmark was transported to the Selhausen test site in Germany and examined on an artificial parcel [18]. In this experiment, Jonard et al. obtained good correlation between the measured and retrieved dielectric constant. However, due to different operating frequencies of the applied devices (a passive radiometer—1.4 GHz and in-situ capacitance sensor—70 MHz) and lack of any reference to SM values the comparison is burdened by significant uncertainties. Hence, the retrieval of SM on organic soil should be thoroughly addressed. Since organic matter in soil can increase saturated soil moisture above 0.6 m^3^m^−3^, which is a boundary of the sensitivity region of SM retrieval algorithms for passive microwave satellites [21], such studies are of significant importance for further development of soil moisture remote sensing methods.

SM retrieval for SMOS level 2 products is performed using the L-band microwave emission of the biosphere model (L-MEB) [22,23]. The full formulation of L-MEB takes into account the roughness expressed by *Q*, *H*, and *N*_p_ parameters (the so-called *QHN* model) [24] and optical depth dependent on the incidence angle expressed by *tt*_p_ [22], where p represents vertical (V) or horizontal (H) polarization. Depending on the land-cover type, those quantities are fixed at certain constant values. However, calibration of those parameters for every SMOS pixel can be very demanding. Therefore it was postulated that in order to lower the number of parameters the vegetation contribution to the microwave emission (optical depth at nadir, *τ*_NAD_) and roughness parameter (*H*_r_) could be combined into *T*_r_ = *τ*_NAD_ + *H*_r_/2 by assuming constant *Q*, *N*_p_, and *tt*_p_ values [25,26,27,28,29,30]. Such simplification (simplified roughness parametrization—SRP) would allow one to make roughness-like parameter independent on predefined land-cover type inside a pixel. It is worth noting that *τ*_NAD_ and *H*_r_ are interdependent and cannot be easily decoupled. Reduction of the amount of semi-empirical parameters from SM retrieval models is the desired direction in their improvement.

In this paper, we present capabilities of equipment located at the Bubnów test site in the monitoring of physical properties of soils together with local climatic parameters for remote sensing studies. The location of the test site has been selected due to the spatial diversity of the area. It is located near a wetland at the border of four land cover types. Therefore its area is characterized by the heterogeneous spatial distribution of SM, organic matter content, and vegetation type. Uniquely, the ESA L-band microwave radiometer (ELBARA) on the Bubnów test site is equipped with an azimuth tracker for automatic mapping of the microwave characteristics of this heterogeneous area while other ELBARA tower radiometers have fixed azimuth direction. It allows studying the SM retrieval models in the presence of the naturally variable amount of organic matter content in soil unavailable on the other test sites. The preliminary SM retrievals are done using the L-MEB–SRP approach [27,28,31]. Previously, it was validated on mineral soils but here we test this approach for also organic soils, so obtained SM values were compared with data recorded by the agrometeorological station and evaluated statistically.

## 2. Materials and Methods

### 2.1. The Bubnów Test Site Description

**Figure 1:**
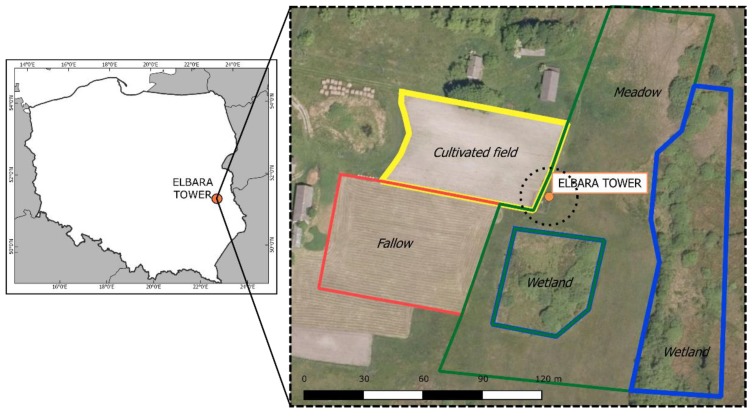
Location of the Bubnów test site. ESA L-band microwave radiometer (ELBARA) III is marked with a circle. The dotted circle indicates a range of the radiometric footprint for the elevation angle 66°.

The test site shown in Figure 1 was located in Eastern Poland, Urszulin commune, near Sęków village, on the border of the Bubnów Wetland (N51.355315, E23.268475). The Bubnów Wetland is a natural swamp belonging to the Polesie National Park (PNP). It is located approximately 170 m above mean sea level with altitude variations less than 5 m. Bubnów area is characterized by low civilization impact, undisturbed hydrological conditions (lack of melioration or irrigation), and mainly traditional agriculture around. In the considered region, there is a warm, temperate climate (the so-called “Land of the Great Valleys” climate), with continental features—short spring and autumn (less than 50 days per year), and long summer and winter (more than 110 days per year).

The bedrock of PNP consists of quaternary or Neogene sediments, deposited in Cretaceous rocks. The flat countryside was formed by the presence of glaciers. The geology of PNP determines the appearance of shallow groundwater, ponds, peat pits and the occurrence of large swamps [32]. The most common type of soils in this area is hydrogenic soils like peat, black earth, soil muck, and glial soil. The peatlands occupy more than 41% of the PNP. The other most common is arenosol [33], which on average consists of 82.9% sand, 15.5% silt, and 1.6% clay. The mean content of soil organic matter in the Polesie region is 3.2% [34].

The test site has an area of approximately 1 ha and is placed near the border of the Bubnów Wetland. The soil characteristics of the test site were performed by laboratory investigation of the soil samples. Over 100 soil samples taken from the Bubnów test site area of approximately 1 ha were examined in a laboratory to determine the contents of sand, silt, clay, and organic matter. Soil examination was performed with the Casagrande areometer method [35]. Organic matter content was obtained using Tiurin digestion and titration method [36]. Point measurements were approximated to continuous spatial distributions using geostatistics (kriging) [37].

### 2.2. Agrometeorological Station Setup

To ensure the availability of suited in-situ data, the Bubnów test site was equipped with two automatic stations: Agrometeorological and thermal-properties station. Stations were located 4–5 m from the ELBARA tower. The agrometeorological station provided standard meteorological data, typical for weather stations, i.e., air temperature and humidity, barometric pressure, wind speed, wind direction, and precipitation amount (including snow). The station measured the energy balance, i.e., incoming short-wave and long-wave radiation versus surface-reflected short-wave and outgoing long-wave radiation. Moreover, the following soil state parameters were measured: Temperature, moisture, salinity, water potential, and radiative temperature of the surface. The station sensors are summarized in Table 1. Measurements of all listed quantities were taken every 10 min and stored in a central unit (CR1000 datalogger, Campbell Scientific Inc., Logan, UT, USA), from where datasets could be downloaded remotely, via GPRS transmission, which was done once per week.

Thermal-properties station provided data on thermal properties and energy fluxes in soil. It measured soil temperature, energy flux, thermal diffusivity, and volumetric heat capacity under the artificial black fallow and grass experimental parcels (Table 1). Measurement of thermal properties requires fluxes stabilization. The time necessary to reach the equilibrium is not constant but depends on the current soil state. Consequently, the time needed for measurements sequence is variable but no longer than 20 min, so there are at least three measurements of all mentioned thermal properties per hour. The acquired dataset was stored in a datalogger.

The presented capabilities of both stations allowed extensive monitoring of the environmental parameters on the test site. For these studies, we used a time-series of topsoil (2.5 cm depth) temperature and moisture (giving approximately 0–5 cm sampled soil layer) measured by Decagon GS3 sensor in one footprint (azimuth 280°, elevation 30°) and local precipitation.

### 2.3. ELBARA III Instrument Characteristic and Data Evaluation

The ELBARA III (third-generation) passive radiometer with a Pickett-type horn antenna is a further development of the ELBARA II [38]. The main improvements in electronics are the new internal temperature control, and two channels per polarization for better radio frequency interference (RFI) detection instead of a single channel used in ELBARA II. The radiometer measures electromagnetic emission at approximately 1.4 GHz. The measurements are acquired with a frequency of 800 Hz by two channels. The voltages measured by the antenna are converted to brightness temperatures *T*_B,P_ for vertical (*T*_B,V_) and horizontal (*T*_B,H_) polarizations in a way shown in Ref. [38]. The horn antenna is situated on the 6.5-m high tower. According to the operating principles of the SMOS satellite, the detecting system of ELBARA radiometer performs angle-resolved measurements by changing the observation angle of the horn antenna. The full range of the elevation angle motion is from 30° to 155°. However, the angles between 30–80° are used for ground emission measurements and 155° for “cold sky” calibration purposes. ELBARA III located on the Bubnów test site has an ability to rotate the horn antenna (changing azimuth) that allows mapping the brightness temperatures around the radiometer. The instrument is situated between four different types of vegetation and land-covers: Meadow, a wetland with temporally open water, fallow, and a cultivated field. The measurements of the brightness temperatures were collected four times a day. Azimuth resolved measurements were taken for every 10° between 0° and 350°. There is a possibility to investigate parameters like the anomalous *T*_B,P_ difference between two channels, kurtosis, skewness, and mean over sigma criterion of the voltage distribution histogram taken from 3200 samples with 800 MHz frequency for two channels per polarization. Additionally, critical values of the internal systems temperature were specified, namely: The active cold source (ACS) and temperature and power controller (TPC) temperatures.

The brightness temperatures acquired by ELBARA III were used as an input to the L-MEB model [22] in order to retrieve SM values on the Bubnów test site area. Fundamentally, the emission of soil can be expressed by [39]:

(1) $$\begin{array}{ccc} & T_{B,P}\left(\theta \right)=e\left(\theta \right)T_{G}, & \end{array}$$

where *e*(*θ*) is the emissivity of soil that can be expressed by the Fresnel reflectivity in the first approximation, and *T*_G_ the thermodynamic temperature of an emitting soil surface. L-MEB model extends this definition by accounting for the roughness parameters and top vegetation layer [22]:

(2) $$T_{B,P}=\left(1-\omega_{P}\right)\left(1-\gamma_{P}\right)\left(1+\gamma_{P}r_{P}\right)T_{C}+\left(1-r_{P}\right)\gamma_{P}T_{G},$$

where *ω*_P_ is the effective scattering albedo for two polarizations (p = H, V), *γ*_P_ is the vegetation attenuation factor, *T*_C_ is the canopy temperature, and *T*_G_ is the ground temperature. The reflectivity of the rough soil surface *r*_P_ is given by the equation:

(3) $$\begin{array}{ccc} & r_{P}\left(\theta \right)=r_{P}^{*}\left(\theta \right)\exp\left(-H_{R,P}\cos^{N_{R,P}}\left(\theta \right)\right), & \end{array}$$

where *H*_R,P_ is the roughness parameter. *N*_R,P_ is introduced to better account angle-dependent and dual-polarization roughness dependencies. The function r*_P_ is the Fresnel reflectivity, which depends on the complex dielectric constant of soil (*ε*_S_) as follows:

(4) $$\begin{array}{ccc} & r_{H}^{*}\left(\theta \right)=\;{\left|\frac{\cos\theta -\sqrt{\varepsilon_{s}-\sin^{2}\theta }}{\cos\theta +\sqrt{\varepsilon_{s}-\sin^{2}\theta }}\right|}^{2}, & \end{array}$$

(5) $$\;\begin{array}{ccc} & r_{V}^{*}\left(\theta \right)=\;{\left|\frac{\varepsilon_{s}\cos\theta -\sqrt{\varepsilon_{s}-\sin^{2}\theta }}{\varepsilon_{s}\cos\theta +\sqrt{\varepsilon_{s}-\sin^{2}\theta }}\right|}^{2}, & \end{array}\;$$

where *ε_s_* is a dielectric constant of soil for a given frequency that can be expressed by water content in soil through a dielectric mixing model. The vegetation transmissivity, *γ*_P_, is basing on the Beer’s law:

(6) $$\begin{array}{ccc} & \gamma_{P}=\exp\left(-\frac{\tau_{P}}{\cos\theta }\right), & \end{array}$$

where *τ*_P_ is the optical depth that can be reduced to nadir (*θ* = 0°) by using:

(7) $$\begin{array}{ccc} & \tau_{P}=\tau_{\mathrm{nad}}\left(tt_{P}\sin^{2}\theta +\cos^{2}\theta \right), & \end{array}$$

where *tt*_P_ for two polarizations accounts for the angle dependence of the optical depth. Equation (2) can be simplified by assuming *ω*_P_ = 0 (for low vegetation covers) [22], *H*_R,V_ = *H*_R,H_ = *H*_R_, and *T*_G_ = *T*_C_ = *T*_GC_ for thermodynamic equilibrium conditions, and then, after considering Equations (2), (3), (6), and (7) the simplified formula reads as:

(8) $$\begin{array}{ccc} & T_{B,P,\theta }=\left(1-r_{G,P,\theta }^{*}\exp\left(-H_{R}\cos^{N_{R,p}}\left(\theta \right)-2\tau_{NAD}\frac{tt_{p}\sin^{2}\theta +\cos^{2}\theta }{\cos\theta }\right)\right)T_{GC}, & \end{array}$$

Further simplification can be done by assuming *N*_r,v_ = *N*_r,h_ = −1 and *tt*_p_ = 1 that leads to the expression:

(9) $$\begin{array}{ccc} & T_{B,p,\theta }=\left(1-r_{G,p,\theta }^{*}\exp\left(-2TR/\cos\theta \right)\right)T_{GC}, & \end{array}$$

where *TR* = *τ*_nad_ + *H*_r_/2. This approach is known as the simplified roughness parameterization [26]. For SM modeling, we have used the Mironov dielectric mixing model with an input of volumetric water content, clay content, and soil temperature [19]. SM retrievals are performed by minimization of the squared difference between measured and estimated brightness temperatures by adjusting the chosen free parameters. 

A parameter that can assess how precipitation influences brightness temperatures and detects water interception by the vegetation is the polarization ratio $PR_{\theta }$ [40,41]. It can be calculated for a given elevation angle θ (for this study, we used the elevation angle *θ* = 60°) using the expression as follows:

(10) $$\begin{array}{ccc} & PR_{\theta }=\frac{T_{B,V,\theta }-T_{B,H,\theta }}{T_{B,V,\theta }+T_{B,H,\theta }}. & \end{array}$$

For bare soil $PR_{\theta }$ increases with increasing soil moisture. Vegetation decreases $PR_{\theta }$ by depolarization of soil emission. A significant drop in $PR_{\theta }$ is observed after rain events caused by interception of water by vegetation. Saleh et al. [42] used $PR_{50}$ to flag data affected by the interception by setting thresholds of $PR_{50}$ ≥ 0.031 and $PR_{50}$ ≥ 0.05 for the high and moderate probability of interception, respectively.

The accuracy of the SM estimation, by means of comparing the retrieved and the measured time-series, was evaluated using the Pearson correlation coefficient (R), bias, root mean square error (RMSE), and unbiased RMSE (ubRMSE) using the equations as follows:

(11) $$R=\frac{\sum_{i=1}^{N}\left(SM{\left(i\right)}_{meas}-\bar{SM_{meas}}\right)\left(SM{\left(i\right)}_{mod}-\bar{SM_{mod}}\right)}{\sqrt{\sum_{i=1}^{N}{\left(SM{\left(i\right)}_{meas}-\bar{SM_{meas}}\right)}^{2}\sum_{i=1}^{N}{\left(SM{\left(i\right)}_{mod}-\bar{SM_{mod}}\right)}^{2}}},$$

(12) $$\;ias=\bar{\left(SM_{mod}-SM_{meas}\right)}\;,$$

(13) $$\;RMSE=\sqrt{\bar{{\left(SM_{mod}-SM_{meas}\right)}^{2}}}\;$$

(14) $$\;ubRMSE=\sqrt{RMSE^{2}-bias^{2}}\;$$

In Equations (11)–(14), *SM*_meas_ and *SM*_mod_ are denoted to the measured and estimated SM values, respectively. The overbar denotes the arithmetic mean of *N* samples in the time-series.

## 3. Results

### 3.1. Bubnów Test Site Soil Characterization

**Figure 2:**
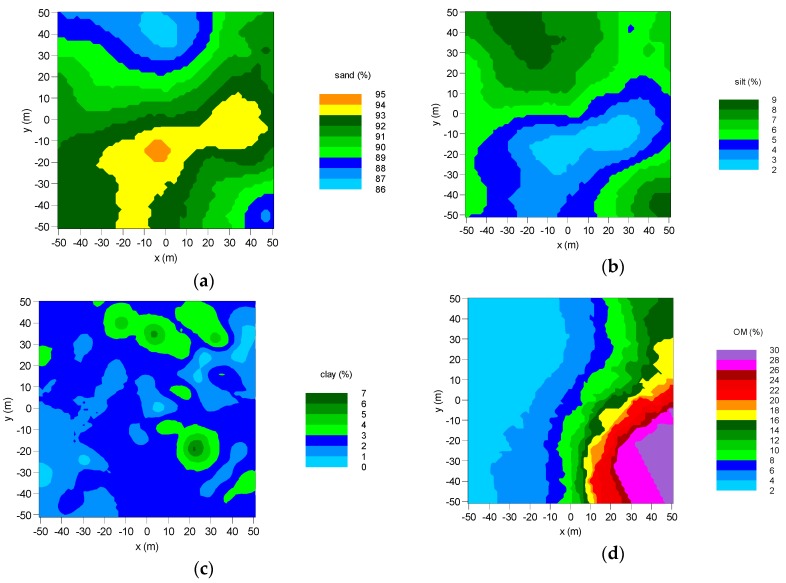

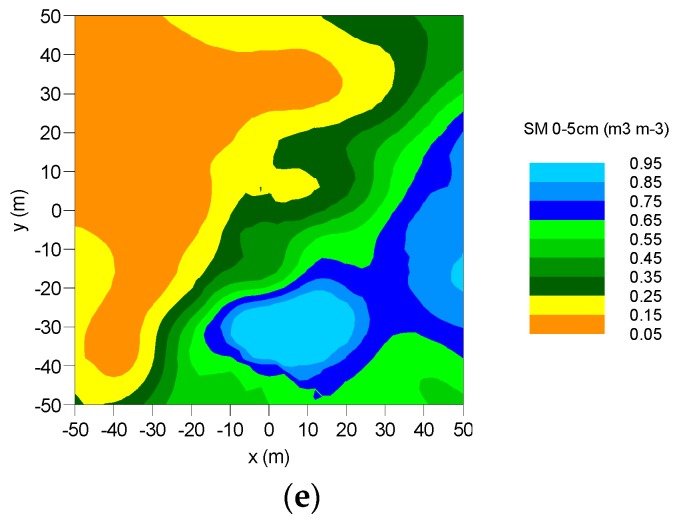
Spatial distributions of (**a**) sand, (**b**) silt, (**c**) clay, and (**d**) organic matter (OM) content in soil on the Bubnów test site. The exemplary distribution of soil moisture (SM) acquired during a field campaign on 18 May 2017 (138 DOY) is shown in figure (**e**). The ELBARA tower is located at (0,0) point of each map.

Spatial distributions of soil granulometric fractions (sand, silt, and clay) and organic matter contents are shown in Figure 2 (together with exemplary soil moisture data). The patterns in Figure 2a,b were similar (with opposite trend) showing that for higher percentages of sand, one could see a decrease in the amount of silt. The highest content of sand with values of 94–95% was observed on the fallow and a part of the meadow area (Figure 1). Adequately, those regions had the lowest content of silt, with a minimum at 2%. Analysis of the spatial distribution of clay content (shown in Figure 2c) revealed its more homogeneous distribution than sand and silt. A large part of the studied area had no more than 2% clay content. However, one could see some single hotspots where the clay content reached 7%. Spatial distribution of OM unveiled a strong spatial anisotropy. The increase in OM content from a value of 2% up to 30% was observed in the east-west direction of the investigated area. Comparing the exemplary distribution of SM (Figure 2e) at the top 5 cm soil layer with OM distribution, one could observe a correlation between these two quantities. The measured SM values vary from 0.1 to 0.95 m^3^m^−3^. The 95% of soil moisture were observed on the meadow and wetland area (saturated peat-bog soil).

### 3.2. Bubnów Test Site Agrometeorological Measurements Time Series

**Figure 3:**
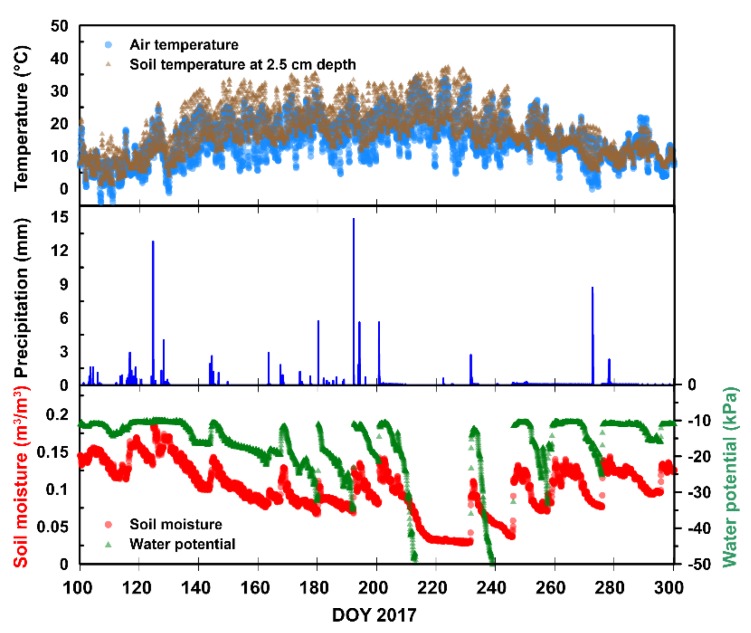
Exemplary meteorological data from the Bubnów agrometeorological station for DOY 100 to 300 of 2017. The figure shows air and topsoil temperature (**top**), precipitation (**middle**), and SM/water potential (**bottom**).

Figure 3 shows data from the agrometeorological station at the test site vs day of the year (DOY). In the analyzed period of 2017, the air temperature fluctuated between −4.8 °C up to 34.7 °C with a mean value of 14.5 °C (top panel of Figure 3). In the case of soil temperature 5 cm below the surface, the maximum recorded value was 37.7 °C, the minimum was 1.7 °C, and the mean value was 17 °C (top panel of Figure 3). In the presented time-series, some significant precipitation events took place with the magnitude above 10 mm (per m^2^ for 10 min measurement) as shown in the middle panel of Figure 3. The lowest soil moisture of topsoil was observed in the middle of August (0.028 m^3^m^−3^). The precipitation events were well reflected in the soil moisture time-series by its increase. The maximum value of SM recorded by the station was 0.21 m^3^m^−3^.

### 3.3. Spatio-Temporal Series of Brightness Temperatures at the Bubnów Test Site

**Figure 4:**
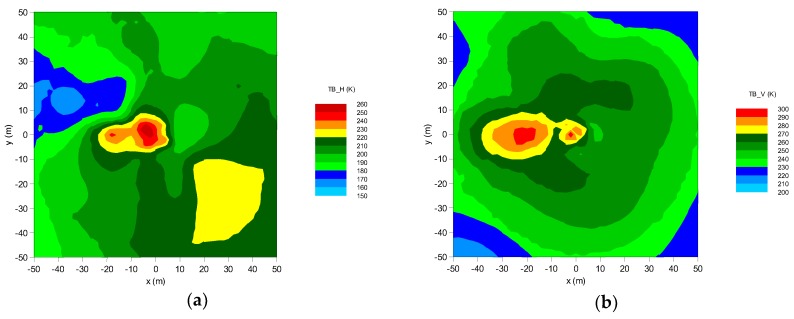
Exemplary brightness temperatures (in kelvins) of the test site, measured by ELBARA (localized in (0,0) point of the plot) for the horizontal (**a**) and vertical (**b**) polarizations. Data collected on 18 May 2017 (138 DOY).

Due to the possibility of rotation ELBARA at different incidence angles and the azimuth tracking capabilities, *T*_B,P_ data might be obtained not only at different distances from the tower but also around it, in footprints containing different vegetation and soil types. During the experiment, data from the area of about 10,000 m^2^ were collected by rotating the ELBARA’s antenna 0–350° in the horizontal and 40–80° in the vertical plane. This type of approach allows combining multiple independent measurements (performed nearly simultaneously) to consistent *T*_B,P_ maps as can be seen in Figure 4. Spatial statistics (kriging) allow correcting blind spots or distortions caused by assembly elements, especially on corners of the ELBARA’s tower. The general purpose of this solution was to enable an analysis of *T*_B,P_ towards obtaining the spatial distribution of soil moisture with time dependency because the maps of the brightness temperatures were taken four times a day.

**Figure 5:**
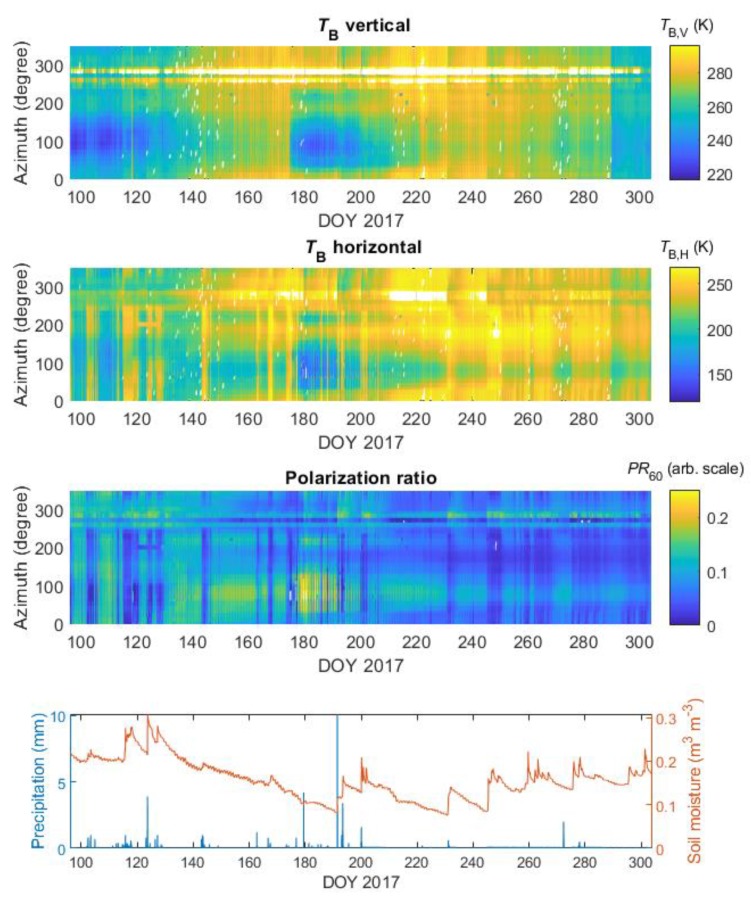
Hovmöller diagrams of brightness temperatures from ELBARA III and the time-series of the SM and precipitation from the agrometeorological station for 2017 (elevation angle 60°). The white color is related to brightness temperatures artificially increased by radio frequency interference (RFI) or other factors.

The spatio-temporal series of L-band brightness temperatures for vertical (*T*_B,H_) and horizontal (*T*_B,V_) for the elevation angle *θ* = 60° are shown as Hovmöller diagrams in Figure 5 [43]. This elevation angle was chosen because most of the collected measurements were not affected by RFI or other factors. Furthermore, *θ* = 60° was at proximity to the Brewster angle, for which changes in *T*_B,P_ were the most sensitive to SM variations (and also near to the maximum sensing angle for SMOS satellite). The values of *PR*_60_ calculated with Equation (10) are also presented. The Hovmöller diagrams were compared to the time-series of the topsoil SM and precipitation depicted in the bottom panel of Figure 5. We analyzed a period from 1 April (91 DOY) to 31 October (304 DOY) of 2017. 

The measurements were taken four times a day, therefore *T*_B,P_ followed diurnal temperature variation (however it was barely visible because of the figure scale). Comparing the time-series of *T*_B,p_ and precipitation, it can be observed that rainfall affected mainly the emission polarized horizontally. We observed an increase in the value of *T*_B,H_, and consequently a decrease PR_60_, during rainfalls. This effect was seen for a majority of azimuths from 0° to 240° and from 310° to 350°. One could see that the time-series values along different azimuths were varying suggesting different sensitivity of the radiometer depending on the cover type within a radiometric footprint. On 174 DOY it could be seen a clear drop of T_B,V_ with a magnitude of approximately 30 K for 80° of the azimuth angle. It was a result of mowing practice on meadow taking place regularly at this time of the year by a farmer. It helps to identify a cover type from the radiometric measurements. Lower brightness temperatures for both V and H polarizations could be seen for meadow (20–240° azimuth angle). Nevertheless, within this range of azimuth angles *T*_B,P_ varied due to spatial heterogeneity of the meadow. The area that the mowing-related *T*_B,P_ drop was not visible was the cultivated field (250–350° azimuth angle). Looking along 100° azimuth after a mowing-related drop of T_B,P_, one could observe that T_B,P_ values restored as the grass was regrowing in time. Brightness temperatures at azimuths 260–290° were increased due to RFI.

**Figure 6:**
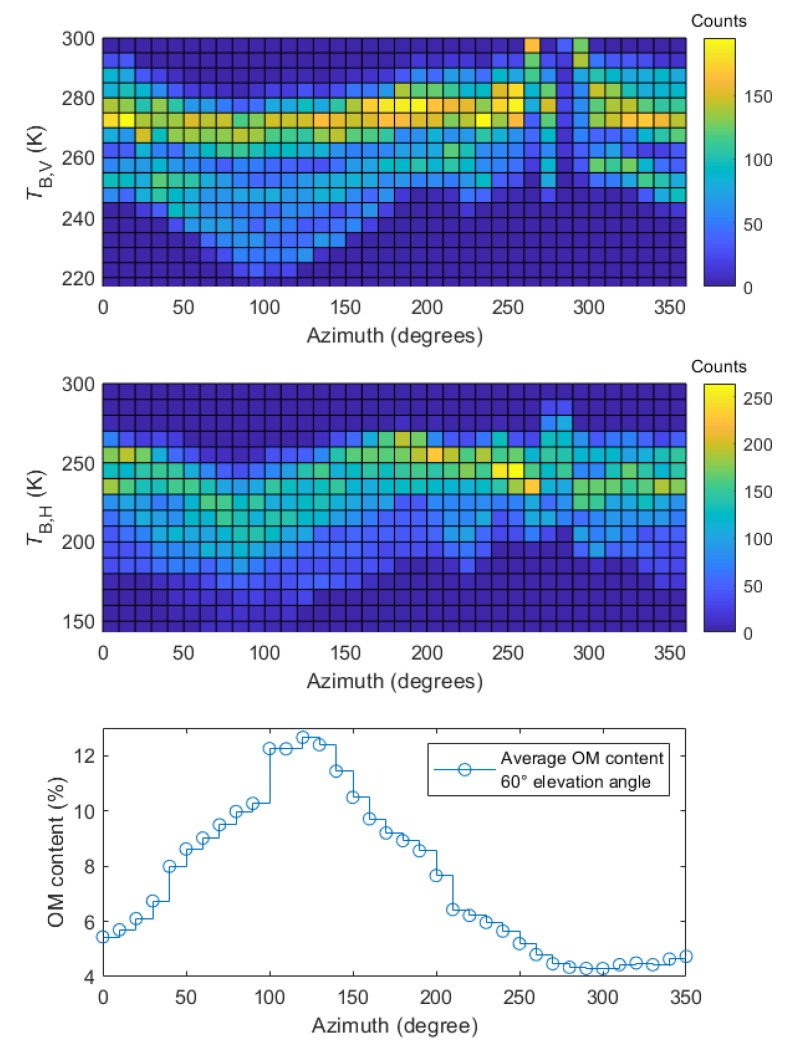
Bivariate histograms of *T*_B,V_ and *T*_B,H_ time-series values vs. the azimuth angle for the analyzed period. The bottom figure shows a distribution of averaged (within the footprint area) OM content for elevation angle 60°.

Figure 6 shows the bivariate histogram analysis of temporal changes of *T*_B,P_ (*p* = H, V) across all the azimuths (the elevation angle 60°) together with the azimuthal distribution of averaged OM content within the areas of certain footprints at the same elevation angle. With increasing azimuth from 0° to 80°, one can observe a shift of the histograms to lower brightness temperatures for both polarizations. In this particular azimuth range, we also observed a broadening of the histogram towards lower *T*_B,P_ values that was more noticeable for V than H polarization. For azimuths from 80° to 200° the histogram shifts back to higher temperatures. Next, for azimuths 260° to 290°, we saw disturbances of histograms related to anomalies in *T*_B,P_, like shifting the peak of the counts distribution to higher temperatures, especially for V polarization. Disturbances in this region can also be seen in the Hovmöller diagrams in Figure 5 and were probably related to RFI from the equipment on agrometeorological station and a lightning protection rod of the ELBARA tower.

### 3.4. L-MEB Modeling Results

Two-parameter (SM, TR) retrieval with L-MEB SRP (Equation (9)) was performed using brightness temperatures at elevation angle 55° and 60° for vertical and horizontal polarizations, acquired during the morning (from 4 a.m. to 8 a.m.) and evening (from 4 p.m. to 8 p.m.) azimuthal scans. To ensure consistency between all used azimuths, we used relatively high elevation angles 55°, and 60° that were also analyzed by other authors in Jonard et al. [18] and Pellarin et al. [44]. For simplicity, we assumed that T_CG_ in Equation (9) could be approximated with the topsoil temperature taken from the agrometeorological station (Figure 3a). The measurements at the agrometeorological station were taken every 10 min. We interpolated the T_GC_ time-series using the b-spline curve in order to estimate *T*_GC_ at the moment of data acquisition by ELBARA. We assumed this temperature was representative for the entire area covered by chosen footprints. As an input to the Mironov dielectric mixing model, we used T_CG_ and clay content [19]. The later parameter was estimated from the continuous clay distribution (Figure 2c) averaged over two (55° and 60°) footprints area. The retrieval results were filtered by *PR*_60_ > 0.05 to avoid interception effects [40], *T*_B,P_ < *T*_CG_ to evade data that were artificially increased by e.g., RFI. Furthermore, data with the sum of squared residuals above 200 (obtained from four data points: *T*_B,V_, *T*_B,H_ for 55° and 60° elevation angles) were filtered out. We assumed that *PR*_50_ > 0.05 condition, used earlier in Saleh et al. [42] was also applicable for the 60° elevation angle.

**Figure 7:**
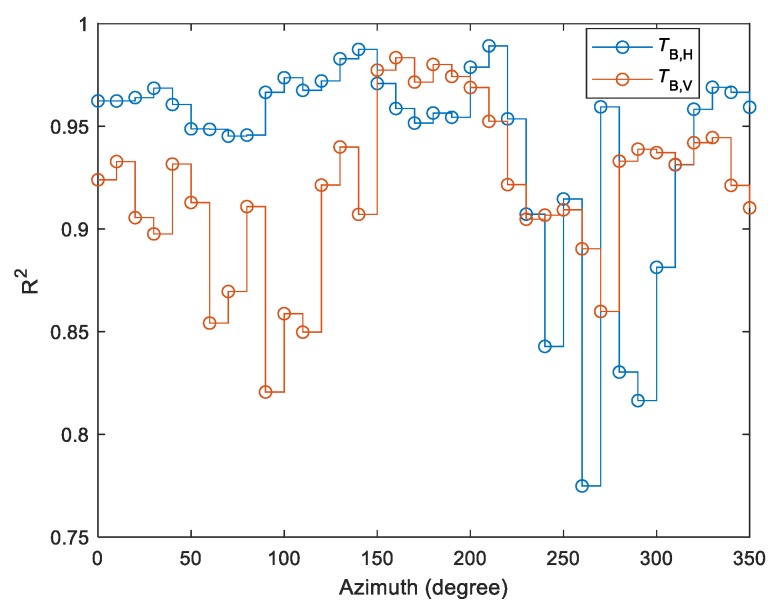
Azimuthally resolved R^2^ values for measured (ELBARA) vs. estimated (L-band microwave emission of the biosphere simplified roughness parametrization—L-MEB SRP) brightness temperatures, at horizontal (H) and vertical (V) polarizations.

The quality of modeling is shown in Figure 7 using the coefficients of determination (R^2^) between measured and estimated *T*_B,P_ separately for two polarizations and all azimuths. The calculated R^2^ values showed a relatively high agreement between estimated and measured data. Nevertheless, the performance of retrievals for H and V polarizations varied spatially, depending on the azimuth.

**Figure 8:**
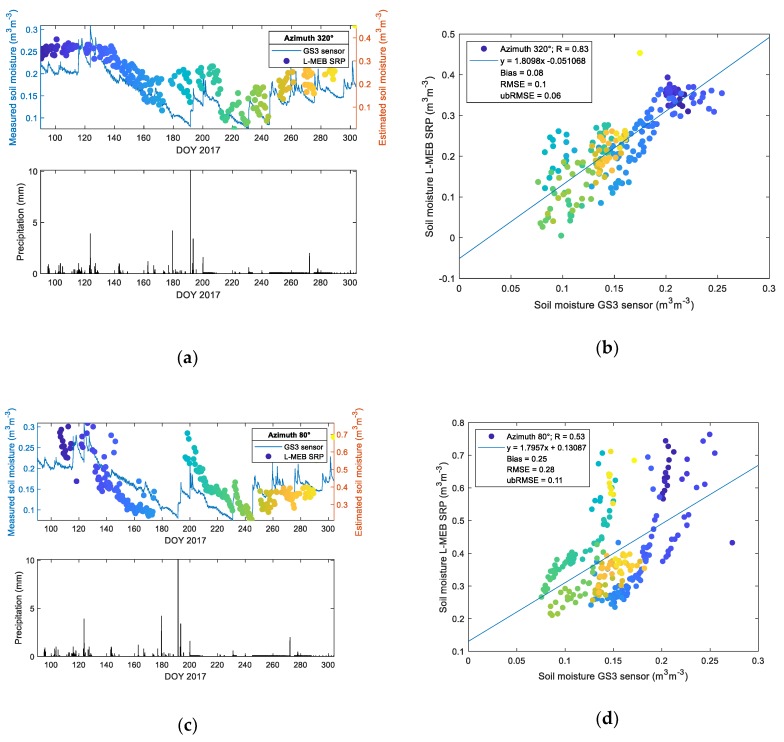
Time-series of estimated (dots) and measured (lines) SM vs. DOY 2017 (top) compared to the precipitation level (bottom) are depicted in panel (**a**) and (**c**). Correlative comparison of estimated and measured SM is shown in panel (**b**) and (**d**). Data are shown for azimuth 320° and 80° for low (4.5%) and high (10%) OM content, respectively. Colors of data-points in figure (b,d) indicate the moment in time for which SM was estimated, also shown in SM time-series in figure (a,c).

To demonstrate modeling results, the SM values retrieved using L-MEB SRP modeling approach compared to SM from the GS3 sensor are shown in Figure 8a–d. It is done for two azimuth angles representing lower (320°—relatively dry cultivated field) and higher (80°—wetland meadow) OM contents (about 4% and 10% on average, respectively). The precipitation level is shown in the bottom figure of the panels (a) and (c). The correlation plots of estimated and measured SM values are depicted in Figure 8b,d, together with results of the statistical analysis of the data by means of R, bias, RMSE, and ubRMSE that is shown in the insets. Coloured points were used to highlight the moment in time for which SM data was estimated.

**Figure 9:**
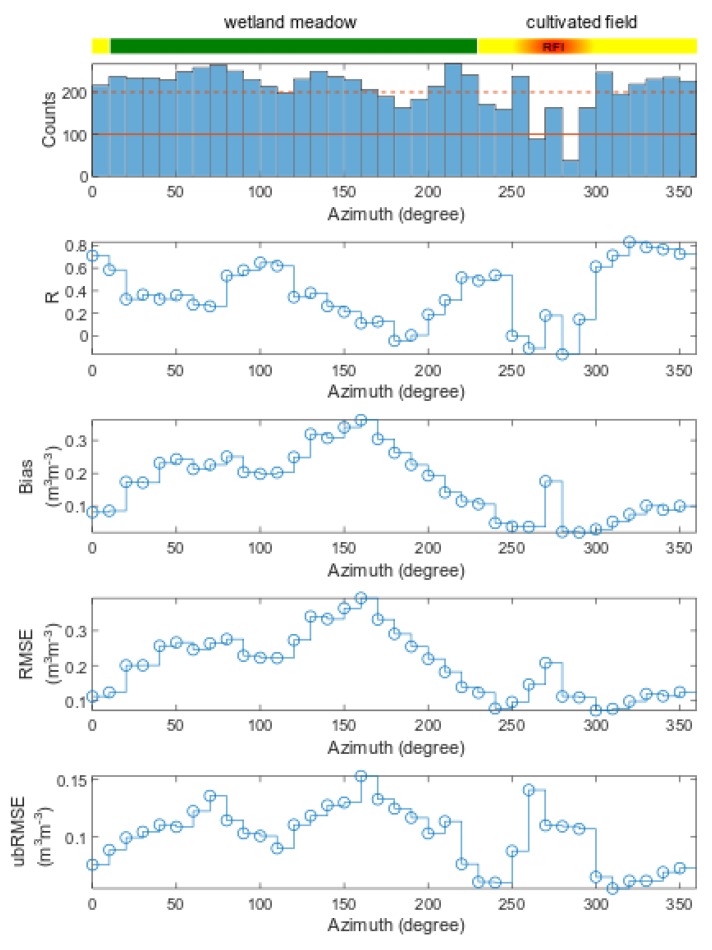
Spatially resolved statistics results for SM (L-MEB SRP modeling) vs. SM probe data. Summary histogram of data counts after data filtering for every azimuth (top) and statistics by means of Pearson correlation coefficient, bias, root mean square error (RMSE), and unbiased RMSE (ubRMSE). Cover type is indicated by a strip on the top of the figure.

As can be seen in Figure 8a,c, the general tendency for revaluation of estimated values is observed. The agreement between analyzed quantities is observed only regarding trends evaluation. Calculated statistics indicate a positive bias demonstrating an overestimation of estimated SM values for both azimuth angles comparing to single point station reference measurements. However, in the case of low organic content, the bias was lower. A similar trend was observed for RMSE and ubRMSE. Summary of statistics for all azimuths is shown in Figure 9.

Due to the pre-analysis criteria, as indicated by the data counts histogram in Figure 9, the amount of data decreased. The most affected azimuths were 260° and 280°, with counts below 100. The second group were azimuth angles with data counts below 200 that were moderately affected. Those two thresholds were marked on the histogram in Figure 9 by solid and dashed lines. Mean R for all azimuths was 0.38 with a standard deviation (σ_R_) of 0.27. The highest R values were calculated for angles 310–350° and 0° that covered the area of a cultivated field. We observed a decrease in the R coefficient on the wetland meadow area. However, a significant drop of the R value was seen between 250–290° azimuth range—the region that was assigned to a cultivated field area. However, we experienced disturbances in that area, the most probably, because of the agrometeorological station parts located therein and influencing *T*_B,P_ values. Mean bias was 0.17 m^3^m^−3^ with σ_bias_ = 0.1 m^3^m^−3^. Unbiasing of RMSE gave its mean value across all azimuths of 0.1 m^3^m^−3^ with σ_ubRMSE_ = 0.03 m^3^m^−3^. The lower values of bias and ubRMSE revealed retrievals over the cultivated field with a minimum of 0.022 m^3^m^−3^ and 0.07 m^3^m^−3^, respectively. The highest bias and ubRMSE was observed for the meadow area at the 160° azimuth angle equal to 0.362 m^3^m^−3^ and 0.153 m^3^m^−3^, respectively.

**Figure 10:**
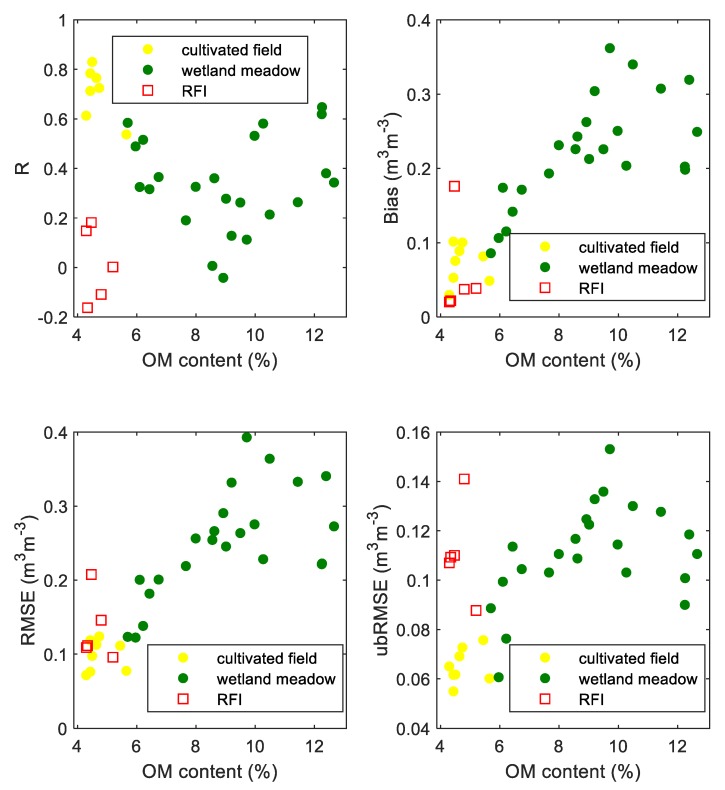
Statistics, by means of R bias, RMSE, and ubRMSE, of SM (L-MEB SRP modeling) vs. SM probe data in relation to OM content for three point-groups representing meadow wetland, cultivated field, and RFI regions.

In Figure 10, statistical measures from Figure 9 were confronted with the OM content for adequate footprints. Colors of data points were analogous to those shown on the strip on top in Figure 9 indicating the land cover type or RFI. In the case of the correlation coefficient (R), one could see that the footprints with low OM content (cultivated field) revealed the highest correlation coefficients. In the low-OM content regions, one could see the values affected by RFI (red squares) gave very low or negative R values. On the areas with OM content above 6%, we observed a wide range of R values from −0.04 (for 9% of OM content) to 0.65 (for 12% of OM content). We observed an increase in bias, RMSE, and ubRMSE with increasing OM content. At about 9%, the values of statistical metrics did not show a noticeable trend, but in general, they were considerably higher compared to the low-OM content cultivated field area.

**Figure 11:**
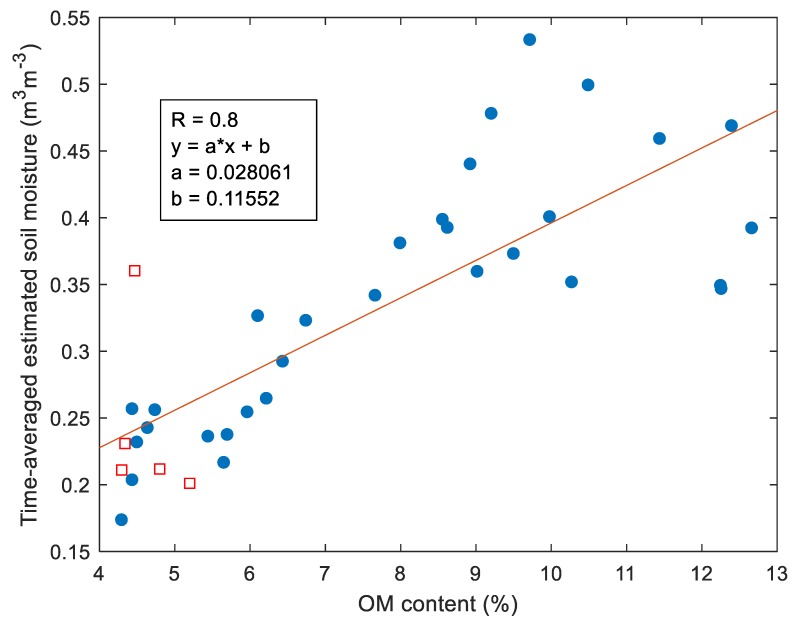
Time-averaged estimated soil moisture retrieved using the L-MEB SRP approach vs. OM content on the adequate azimuths.

In Figure 11, the average values of estimated SM time-series for every azimuth confronts the OM content on the investigated footprints. The correlation coefficient of such a comparison was 0.8 (without taking into account RFI points) indicating a strong relationship between those two quantities. 

## 4. Discussion

In this study, we showed the capabilities of the Bubnów test site for microwave remote sensing model calibration and validation studies. In particular, we were able to capture spatio-temporal variability of *T*_B,P_ of the diversified area with a variable OM content. We also modeled the SM with the L-MEB SRP approach and compared to the SM data from the agrometeorological station. 

The Bubnów site’s soil was characterized by means of the spatial distribution of granulometric composition, organic matter content, and soil moisture to support continuous spatio-temporal studies on the L-band microwave emission with the ELBARA III radiometer. As seen in Figure 2, sand, silt, and clay contents varied from 86% to 95%, 2% to 9%, and 0% to 7%, respectively. According to USDA soil classification, the soil on the test site area is loamy sand [45]. Exemplary distribution of measured SM, shown in Figure 2e, could not be explained only by the variations in granulometric composition. The spatial variability of SM could vary from 5% to 95% in this small area due to changes in OM content, which distribution is shown in Figure 2d. Such a change in OM content is essential for the Bubnów test site for studying microwave emission from the soil with different water contents.

Hovmöller diagrams expressing spatio-temporal series of *T*_B,P_ revealed azimuth-specific behavior depending on the land cover type. One could see a clear interception-related increase of *T*_B,H_, and consequently decrease in *PR*_60_, for almost all azimuths, similar to previous reports [40,41]. This effect introduces uncertainties to SM retrievals. Mowing of grass on the meadow on 174 DOY, shown in Figure 5, caused a decrease in *T*_B,H_ and *T*_B,V_ and increase of the *PR*_60_. A similar effect was observed in Ref. [46]. 

A different perspective on *T*_B,P_ values gives bivariate histogram of the time series, shown in Figure 6. The behavior of *T*_B,P_ at azimuths 0° to 200° compared to OM content distribution indicates their negative correlation. Namely, with increasing OM content (and consequently SM) in soil *T*_B,P_ tended to decrease by a dozen of kelvins at its minimum at approximately 80° of the azimuth angle. We suggest that this could be an effect connected with a change in soil’s structure, giving a negative contribution to a roughness parameter. Soil moisture modifies a roughness parameter by the so-called “dielectric roughness”. As soil moisture increases dielectric roughness decreases. Therefore, in the case of wet conditions, the observed decrease in *T*_B,P_ is connected with the smaller microwave sampling depth [47,48,49], which is in agreement with soil moisture, and OM distributions on the Bubnów test site demonstrated in Figure 2 and Figure 6 (bottom), respectively. One could relate the simultaneous increase of the SM and OM content by moving from 0° towards 80° azimuth angle together with a downward shift of histograms of *T*_B,V_ and *T*_B,H_ in Figure 6 (and opposite trend after 90°).

Pre-filtering data by the PR_60_ value and setting up a threshold on the sum of squared residuals of fit was performed in order to provide the high accuracy of L-MEB SRP retrievals by means of estimated vs. measured T_B,P_ depicted in Figure 7. It enabled getting rid of interception and other effects that affected the retrievals. The results of SM retrievals are summarized in Figure 9 by azimuthally resolved statistics. The example of time-series and SM correlative plots (estimated vs. measured) for two cases of lower and higher OM contents are shown in Figure 8. It is worth emphasizing that the comparisons were made using a single point SM data from the agrometeorological station representing data from relatively low OM content with data from all the azimuths with a variety of soil attributes (Figure 2).

The highest R coefficients represented the cultivated field area between azimuths 230° to 350° and 0° with relatively low OM content. However, between 250° and 290°, one could see a drop of R. The reason may lie in the components of the agrometeorological station elements within footprints and influencing *T*_B,P_ values by RFI. In this area, we saw also an increased bias for the 270° azimuth angle and RMSE for 260° and 270°. However, the disturbances were well emphasized in ubRMSE, where one could see its increase between 250° and 290°. A similar cover type above 300° kept a relatively high R (0.61–0.83), and low bias (0.04–0.1 m^3^m^−3^), RMSE (0.07–0.124 m^3^m^−3^), and ubRMSE (0.05–0.075 m^3^m^−3^). The result of ubRMSE for the cultivated field was slightly above 0.04 m^3^m^−3^—the expected value for the SMOS satellite mission performance. A relatively high level of bias in this area could be a result of a small amount of OM estimated from geostatistics (Figure 2d and Figure 6). On the other hand, the bias of the retrieved SM values could be caused by the L-MEB model itself, but the exact source is unknown [13]. It is worth noting that at a global scale L-MEB SRP underestimates SM values [26].

The wetland meadow region revealed distinctly lower R coefficients compared to the cultivated field. It could be related to the spatial distribution of the OM content around the radiometer (the bottom of Figure 6) resulting in spatially different wetting/drying dynamics of footprints comparing to soil from the station, and consequently, the different character of microwave emission due to changing conditions. One can see a systematic increase of bias and RMSE up to 0.36 m^3^m^−3^ and 0.39 m^3^m^−3^, respectively, moving from 10° to 160° azimuth angle. It means that the retrieved SM values were overestimated compared to the station’s SM measurements. However, this increase could also be explained by the OM distribution and consequently, real SM distribution on the test site. Nevertheless, the peak of the OM content was on the 120° azimuth angle comparing to the peak of bias at 160°. This particular shift could be explained that the radiometer could eventually pick some border parts of wetland (with temporarily open water and different vegetation cover comparing to meadow). Despite generally low R on the wetland meadow area, the variating bias indicates that the retrieval model generally follows the mean level of soil moisture depending on the azimuth. The results revealed a high bias at azimuth angles with relatively high OM content areas. A more comprehensive view on the influence of OM content at the footprints on statistical metrics is shown in Figure 10. In particular, one could see an increasing bias, RMSE, and ubRMSE with OM content. It could be seen that 7% OM content was enough to cause a meaningful increase in bias, RMSE, and ubRMSE. However, the trend was not straightforward for R values. We observed a decrease in R with increasing OM content (up to 9%). For OM contents >9%, the R values were more spread. Figure 11 shows time-averaged estimated SM values compared to OM content at the relevant azimuths. It showed an increasing dependence with a strong correlation of R = 0.8, reflecting the general truth that OM increases the water holding capacity in soil. Interestingly, in Figure 11, one could notice that below 9% OM content, the SM vs. OM relation seemed to be quasi-linear, while above 9% was rather spread. Figure 11 is also very similar to the bias and RMSE plots from Figure 10. We showed the results that were not validated by the ground-truth measurements, but rather a demonstration of qualitative correctness. It is worth noting that those results were obtained with the L-MEB SRP approach that had no parameters for calibration, and the applied dielectric mixing model was based on clay content.

Finally, we discussed some aspects of measurement of the L-band microwave emission and modeling of SM of rich-organic soil on the Bubnów test site. There is a need to further investigate the influence of the OM on the microwave emission of soil towards the improvement of satellite remote sensing methods. In future studies, we plan to study the L-band emission from organic soil at the Bubnów test site and compare the results with the spatial distribution of SM measurements collected from in-situ probes or during the field campaigns.

## Figures and Tables

**Table 1:** The measurement capabilities of the stations on the Bubnów test site.

Measured Features	Number of Sensors	Additional Information
Precipitation	1	Rain gauge with heater (in order to melt snow).
Air temperature	1	Placed 2 m above ground (meteorological standard).
Air humidity	1	
Wind speed	1	-
Wind direction	1	-
Barometric pressure	1	Standard.
Energy balance	1	Measure incoming and reflected radiation, both in short- and long-wave.
SM, temperature and salinity	10	Five sensors placed in artificial black fallow (two at 2.5 cm depth, two at 10 cm, and one at 20 cm). The same configuration for another five sensors placed in grass parcel.
SM (profile)	2	Profile probes with sensing elements at 10, 20, 30, 40, 60, and 100 cm depth; one profile probe is placed in artificial black fallow, the second in grass parcel.
Soil temperature (profile)	7	Profile probes with sensing elements at 1, 1.5, 2, 3, 4, 6, 8, 10, 12, 16, 20, 24, 32, 48, 64, and 100 cm.
Soil water potential and soil temperature	10	Five sensors placed in artificial black fallow (two at 2.5 cm depth, two at 10 cm, and one at 20 cm). The same configuration for another five sensors placed in grass parcel.
The radiative temperature of the soil surface	2	One pyrometer is permanently aimed at a grass parcel. The second is moving with ELBARA cone, measuring the radiative temperature at measured areas (footprints).
Soil temperature (profile)	2	Profile probes with sensing elements at 1, 2, 4, 8, 12, 16, 24, 32, 48, 64, and 96 cm; one placed in black fallow, the second in grass parcel
Energy flux in soil	8	Four sensors placed in artificial black fallow (at 2, 6, 14, and 28 cm depth). The same configuration for another four sensors placed in grass parcel.
Soil thermal diffusivity and volumetric heat capacity	6	Three sensors placed in artificial black fallow (at 4, 14, and 28 cm depth). The same configuration for another three sensors placed in grass parcel.
